# Botanical and Traditional Uses and Phytochemical, Pharmacological, Pharmacokinetic, and Toxicological Characteristics of *Ziziphi Spinosae Semen*: A Review

**DOI:** 10.1155/2020/5861821

**Published:** 2020-07-10

**Authors:** Su-Rong He, Chong-Bo Zhao, Jing-Xia Zhang, Jing Wang, Bo Wu, Chun-Jie Wu

**Affiliations:** ^1^College of Pharmacy, Shaanxi University of Chinese Medicine, Xianyang 712046, China; ^2^College of Pharmacy, Chengdu University of Traditional Chinese Medicine, Chengdu 611137, China

## Abstract

*Ziziphi Spinosae Semen* (ZS, the seeds of *Ziziphus jujuba Mill*. var. *spinosa* (Bunge) Hu ex H. F. Chou) is used as a traditional Chinese medicine referred to as Suan zao ren (酸枣仁). This paper aims to provide a systematic review of its traditional uses and its botanical, phytochemical, pharmacological, pharmacokinetic, and toxicological characteristics. The future development and research prospects for *ZS* have also been discussed in detail. To date, over 150 compounds have been identified in this plant, including terpenoids, alkaloids, flavonoids, fatty acids, volatile oils, polysaccharides, and others. Both extracts and purified compounds have excellent biological activities, especially sedative and hypnotic effects. Other effects include ameliorating effect of learning and memory, anti-inflammation, antioxidation, blood pressure and lipid lowering, antiaging, and antitumor effects. Thus, this traditional Chinese medicine can be used to treat many diseases such as insomnia, forgetfulness, headaches, and dizziness. Although many of the traditional uses of ZS are well established, the relationship between structure and function still needs to be further studied. In order to better pave the way for research and the establishment of quality control standards for ZS, it will be very important to elucidate its pharmacological mechanisms of action and explore new clinical effects.

## 1. Introduction

*Ziziphi Spinosae Semen* (ZS) has a long history as an effective traditional Chinese medicine [1]. ZS mainly grows in Asia, Europe, Australia, and especially in the inland areas of northern China. ZS tree is a relatively common wild resource in northern China. There has been a saying that “thorns are everywhere” since ancient times. For this reason, the tree of ZS was also commonly known as a “thorn” in ancient China [2].

Presently, more than 30 prescriptions for ZS are listed in the Pharmacopoeia of the People's Republic of China (ChP). These prescriptions have been used to treat palpitations, insomnia, dizziness, headache, nausea and vomiting, coughs, depression, anxiety, and other diseases. More than 150 compounds have been isolated from ZS, including terpenoids, alkaloids, flavonoids, fatty acids, volatile oils, polysaccharides, and some inorganic compounds [3–5]. Moreover, numerous researchers have been found that ZS has a wide range of pharmacological activities including ameliorating effect of learning and memory, anti-inflammatory, antioxidant, antihypertensive, hypolipidemic, antiaging, and anticancer properties [5, 6]. ZS is mainly used for medicinal purposes.

In this review, we collected the relevant literature, ancient books, and Ph.D. and MSc dissertations related to ZS from a range of scientific databases including PubMed, Baidu Scholar, Vepsa, Wanfang Med Online, CNKI, and others and have summarized the various aspects of ZS, such as its traditional uses and its botanical, phytochemical, pharmacological, pharmacokinetic, and toxicological characteristics. Finally, current problems and research directions for ZS have also been discussed in this paper.

## 2. Botanical Characteristics

**Figure 1:**
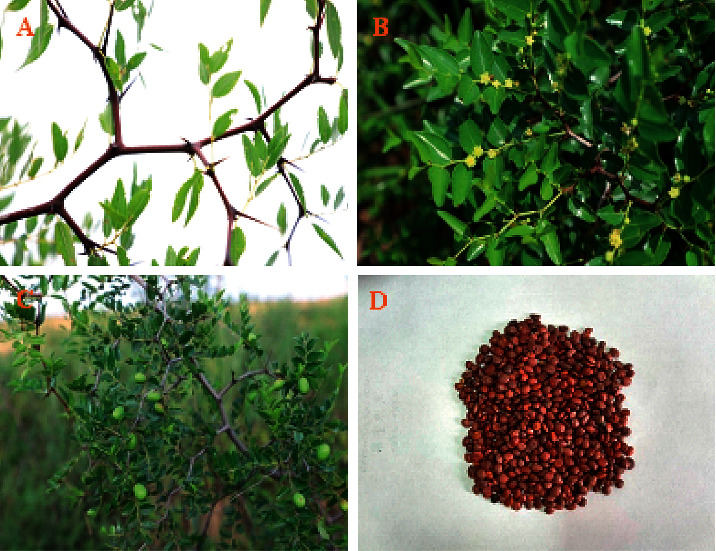
The stem (a), the leaves and flowers (b), the fruits (c), and the seeds (d) from ZS trees.

*Ziziphus jujuba* Mill. var. *spinosa* (Bunge) Hu ex H. F. Chou (Rhamnaceae) is a deciduous shrub or small arbor approximately 1–3 m in height (Figure 1). It is a kind of drupe; seeds (ZS) are often used as medicinal parts in traditional medicine.

Older branches are brown, and younger branches are green. The stems are branched, with straight or curved prickles. The leaves are typically 2.5–5 cm long and 1.2–3 cm wide, with the color varying from light to dark green. The flowers are small and yellow-green with 2∼3 clusters of leaf axils. The calyces contain five lobes and an oval triangle. Every flower has five petals and five stamens. The petals are small and yellowish green in color and alternate with the sepals. The stamens and petals grow opposite to each other. The flower disks are very evident and contain 10 lobes. The ovary is oval and is buried in the flower disk and has two lobes. The fruits are oblate or oval, 6–8 mm in diameter, and 3 mm in thickness. Before maturity, the surface of the fruit is smooth and green colored, whereas the mature fruit is purplish red colored. One side of the fruit is flatter than the other and has a slightly uplifted longitudinal line in the center; the other side appears as a bulge. The fruit tastes sour-sweet and has a light odor. The fruits are harvested between September and October. After harvest, they are typically soaked overnight and the flesh is removed before separating the seed, after which the core shell is crushed and the kernel is removed and sun-dried [5, 7]. Although ZS tree is native to China, it is now cultivated in other Asian countries, Europe, and Australia. It is widely distributed in the Chinese provinces of Shandong, Shaanxi, Hebei, Liaoning, Henan, Shanxi, and Gansu, among which Hebei and Shaanxi province are the largest producers and are famous for their high production and quality [1, 8, 9].

## 3. Traditional Uses

The cultivation of ZS tree has a history of nearly 2000 years. Particularly in the Loess Plateau of China, clusters of ZS tree have been growing since ancient times, and there is one of the oldest ZS trees which has been growing for more than 2000 years in Shanxi Province of China. ZS can be used as effective medicine to nourish the brain and promote hemogenesis, and it is the first type of fruits that can be used for medicinal purposes and as a food that has been promulgated by the Ministry of Health [10]. ZS was first described in “*Shen Nong's herbal classic*” (the earliest Traditional Chinese Medicine (TCM) monograph during the Eastern Han Dynasty) under the name “*Suanzao.*” Medical books about ZS first appeared in “*ShanghanZabingLun*” (Eastern Han Dynasty) which was famous for the decoction of spine date seeds, and “*Suanzaoren*” was first listed in “*Materia Medica ChengyaBanju.*” Therefore, ZS has more than 1700 years of medicinal history. In “*Chinese Materia Medica,*” ZS trees were referred to as “*Qi*,” “*Jing*,” “*Shanzao*,” and “*Yezao*” [11]. In ancient China, people discovered its ability to eliminate disease and prolong life, and it was used as herbal medicine in successive dynasties after the Tang Dynasty. “*Ben Cao Shu Gou Yuan*” stated that ZS trees were used as medicines in“*Shen Nong's herbal classic*” (Qing Dynasty), but, currently, the seeds (ZS) are used as a traditional Chinese medicine [7]. “*Shen Nong's herbal classic*” gave ZS a top grade and noted that it is an effective medicine for regulating the five Zang organs (heart, lungs, kidneys, liver, and spleen) and longevity [8]. It is usually stir-fried, especially for the treatment of sedation and hypnosis [9]. “*Ben Cao Bian Du*” noted that fried ZS can cure insomnia of colic asthenia and raw ZS can cure sleepiness caused by colic heat. In addition, according to “*Bie Lu*” (Western Han Dynasty), ZS could treat cold sweat and polydipsia. “*Ben Cao Zai Xin*” described the qi and hidroschesisas tringing function of ZS, in addition to benefiting ambition and nourishing the ear and eye [11]. It also was used to treat painful limbs and dampness syndromes in “*Shen Nong's herbal classic*.”

Currently, ZS is an important Chinese medicine for the treatment of insomnia clinically. It can also be used to treat palpitations, dizziness, dietary intolerance, nausea and vomiting, coughs, and other diseases. Spine date seed decoction, a classical clinical prescription, can be used to treat many diseases, including psychiatric disorders such as depression and anxiety, neurological disorders such as headache and vertigo, and circulatory diseases [12]. There are many prescriptions recorded in the ChP such as pills, tablets, capsules, and granules, with pills and tablets being the most commonly used. Among the prescriptions for palpitation and insomnia, ZS is the main medicine, while the prescriptions for other diseases are relatively minor. Moreover, the high nutritional value of ZS makes it indispensable in the health beverage industry. Beverages made from ZS are considered nourishing and strengthening. Common drinks made from ZS include wine, juice, and syrup [13].

## 4. Phytochemistry

**Figure 2:**
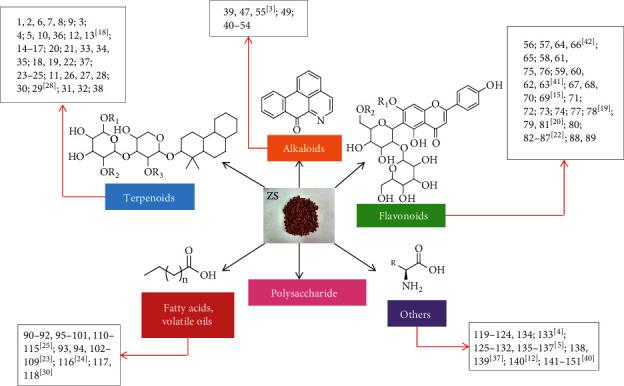
The structure diagram of all the compounds in ZS.

**Figure 3:**
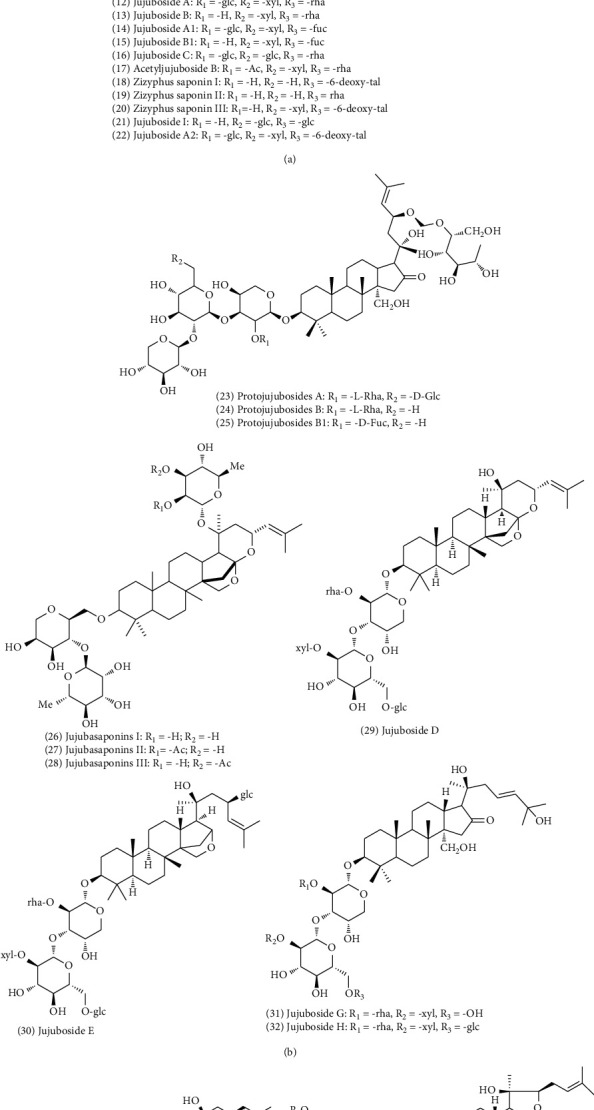
Chemical structures of terpenoids in ZS.

**Figure 4:**
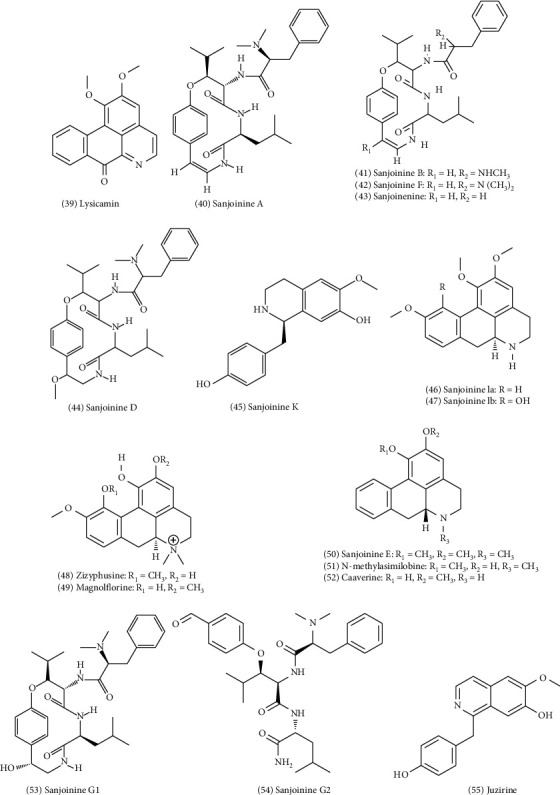
Chemical structures of alkaloids in ZS.

**Figure 5:**
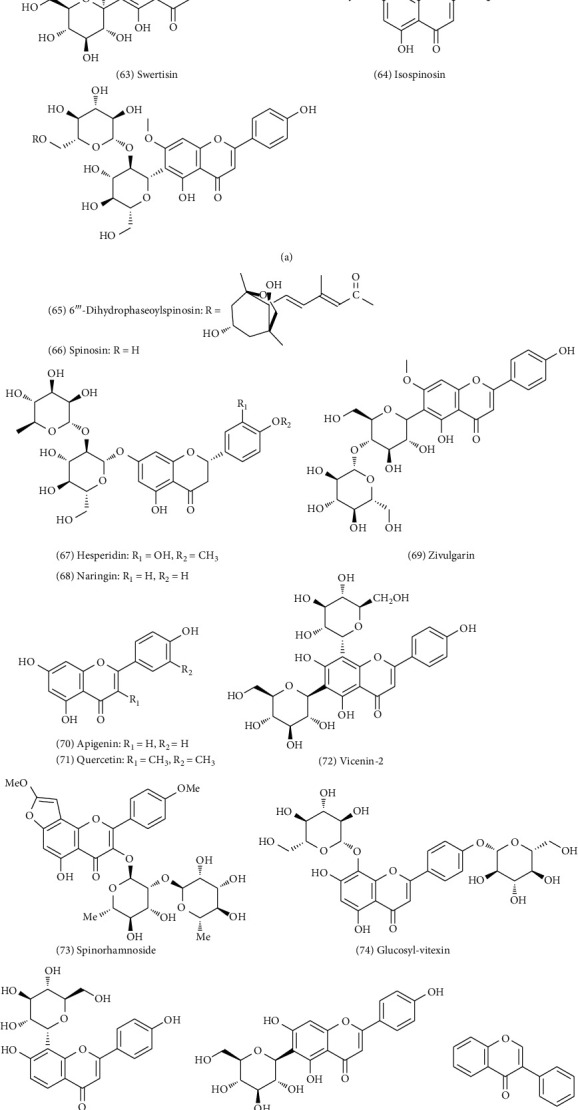
Chemical structures of flavonoids in ZS.

**Figure 6:**
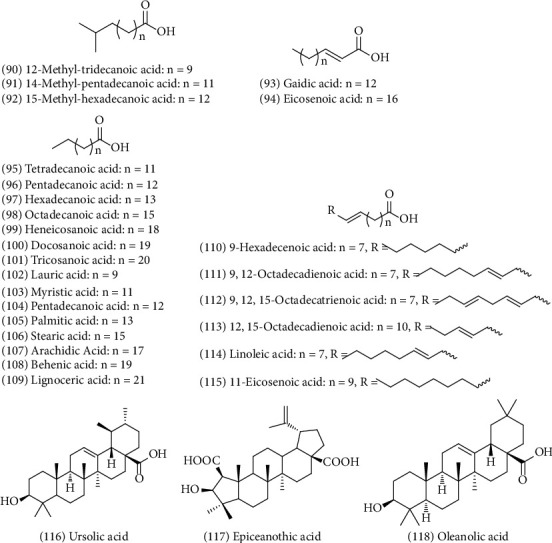
Chemical structures of fatty acid and volatile in ZS.

**Figure 7:**
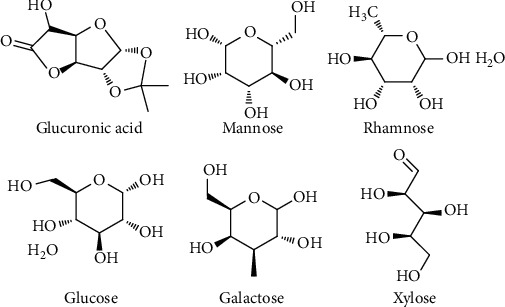
Monosaccharides included in the purified ZS polysaccharide.

**Figure 8:**
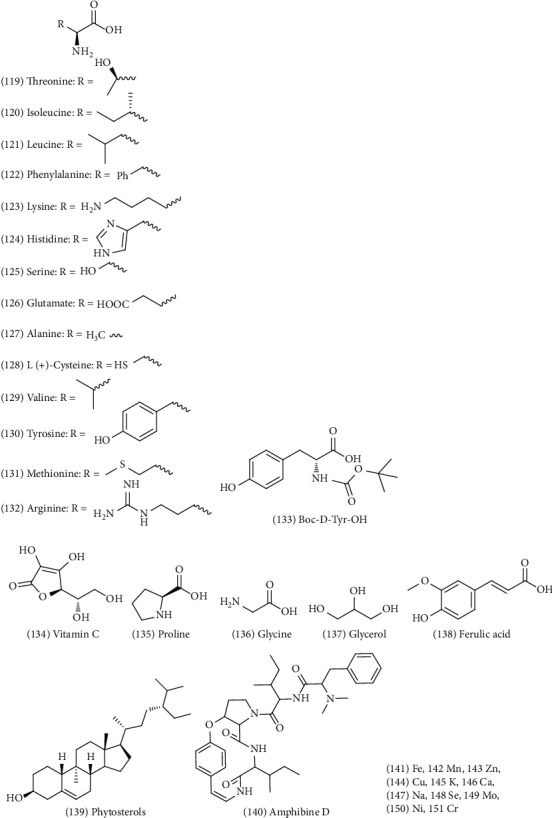
Other chemical structures in ZS.

The chemical composition of ZS is extremely complex. It has been studied for more than 30 years. Over 150 different chemical components, including terpenoids, alkaloids, flavonoids, fatty acids, volatile oils, and polysaccharides, have been isolated and identified (Figure 2). The specific chemical structures are shown in Figures 3–8. The chemical compositions and corresponding structures of ZS have been comprehensively discussed in this paper with references provided for further study and some ideas for future research directions.

### 4.1. Terpenoids (1–38)

The main chemical compositions in ZS are saponins such as jujuboside A (No. 12), jujuboside B (No. 13), and jujuboside C (No. 16), a triterpenoid, which are closely related to the active ingredient saponin in *pseudo-ginseng* and *ginseng*. It is generally believed that it is an effective sedative component of ZS and has a strong central inhibitory effect. Jujubosides have been extensively studied. Otsuka et al. first obtained jujuboside A and jujuboside B from a methanol extract of ZS in 1978 [14]. Following this, several other terpenoids were isolated and identified, and currently more than 30 terpenoids have been identified. Their nuclear parents are all of the damaranen type, which is derived from jujubogenin [4]. Lupinane, oleanolic, and ceanothane triterpenoids are the three main triterpenoids found in ZS [3]. The chemical constituents of terpenoids and their corresponding structures are shown in Figure 3.

### 4.2. Alkaloids (39–55)

The research level of alkaloids in ZS is far less than that of terpenoids. Two alkaloids, lysicamine and juzirine, were first isolated from ZS by Yin et al. in 1997 [3]. Following this, cyclopeptide alkaloids, such as sanjoinine G_1_ (No. 53) and G_2_ (No. 54), and aporphinoid alkaloids such as sanjoinine E (No. 50), K (No. 45), la (No. 46), and lb (No. 47), were discovered [4]. Sanjoinine E is also known as nuciferine, sanjoinine la is also known as nornuciferine, sanjoinine lb is also known as norrisocorydine, and sanjoinine K is also known as coclaurine [5]. In addition, several other alkaloids have also been found in ZS. The chemical constituents of the alkaloids and their corresponding structures are shown in Figure 4.

### 4.3. Flavonoids (56–89)

Flavonoids are one of the main active constituents in ZS and all of them are flavone-C-glycosides. Won et al. first isolated spinosin (No. 66) from methanol extract of ZS in the late 1970s [15]. Following the determination of the structure of flavone-C-glycoside, an increasing number of flavonoids have been isolated and identified. In 1986, zivulgarin (No. 69), a new flavonoid compound was isolated from ZS by Zeng et al. [16]. More recently, eight new compounds were isolated for the first time from ZS by Wan et al. in 2008 [17]. Cao et al. isolated seven compounds from ZS, among which quercetin (No. 71) was isolated for the first time [18, 19]. Currently, more than 20 types of flavonoids have been isolated and identified as the main components responsible for treating sedation and hypnosis. The chemical constituents and their corresponding structures are shown in Figure 5.

### 4.4. Fatty Acids and Volatile Oils (90–118)

ZS oil is stable on oxidation and has oil content as high as 31.8% and total vitamin E content of 0.1867 mg/g [20]. In 2006, Wang et al. extracted ZS by Soxhlet extraction to determine fatty acids. The fatty acids in the oil were esterified by sodium hydroxide-methanol and then analyzed by gas chromatography-mass spectrometry (GC/MS). They separated and identified 17 different types of fatty acids from ZS, accounting for 95.33% of the total fatty acids. Unsaturated fatty acids account for 80.20% and saturated fatty acids account for 15.13% of the total fatty acids [21]. In addition, ZS contain about 32% fat oil. Che et al. extracted 21 different types of fatty oils from ZS using supercritical CO_2_ extraction [22]. In 2001, Chen et al. first applied supercritical fluid extraction technology to extract fat oil from ZS and as a result identified 12 fatty acids [20]. Lu et al. showed that ZS oil is mainly composed of six fatty acids, among which oleic acid, linoleic acid, and linolenic acid had the highest contents of 49.1%, 26.0%, and 4.1%, respectively. In addition, the oil of ZS contains a small amount of lauric acid, palmitoleic acid, and docosahexanoic acid [23]. A summary of the fatty acids and volatile oils found in ZS is shown in Figure 6.

### 4.5. Polysaccharides

ZS polysaccharide is a kind of polysaccharide extracted from ZS. Previous studies have shown that ZS polysaccharide had obvious anti-inflammatory and immune-regulating biological activities [24, 25]. In 2008, Lin et al. showed that the optimal conditions for perfect yield (1.05%) of polysaccharide extracted from ZS with ultrasound assisted extraction method were extraction temperature 52.5°C, extraction time 21.2 min, extraction power 134.9 W, and ratio of liquid to solid 26.3 mL/g. HPLC-ELSD method determined that ZS polysaccharide mainly contains two kinds of polysaccharides with molecular weights of 10000 kDa and 2.34 kDa, respectively. In addition, the purified polysaccharide contains glucuronic acid (0.89%), mannose (15.42 ± 0.08%), rhamnose (18.36%), glucose (0.20%), galactose (35.49%), and xylose (29.33%) (Figure 7) [26]. In the same year, they also found that ZS polysaccharide decolorization parameters with D101 macroporous adsorption resin (2 mg/mL for the sample concentration, 3.5 mL for the volume, and 4 BV/h for the elution flow rate) is the best decoloration method. Under the condition, the values of polysaccharide decolorization rate and retention rate were 61.32% and 87.05%, respectively [27].

### 4.6. Other Constituents (119–151)

ZS also has various trace elements and eight amino acids that are essential to humans [17, 28]. Non ribosomal peptides [29], ferulic acid (No. 138), phytosterols (No. 139) [30] and vitamin C (No. 134) have also been found. In addition, some other compounds of ZS have been identified [31]. A summary of the other chemical compounds found in ZS is shown in Figure 8.

## 5. Pharmacology

ZS-derived extracts are pharmacologically active and exert many effects, including that on the central nervous system and the cardiovascular system. The main pharmacological effects of ZS include sedation and hypnosis, ameliorating effect of learning and memory, anti-inflammation, antioxidation, anticonvulsion, blood pressure lowering, lipid lowering, antiplatelet aggregation, enhancement of immune function, antihypoxia, antimyocardial ischemia, antiarrhythmia, and antiaging. In this section, the different pharmacological activities of ZS have been introduced and analyzed. A summary of pharmacological effects of ZS is shown in Table 1.

### 5.1. Effect on the Central Nervous System

Wang et al. studied the effects of ZS flavonoids (0.1 g/kg) on improving the learning and memory ability in memory-impaired mice by using dark-avoidance test and maze test. The data showed that flavonoids could prolong the latent period of mice, reduce the erroneous times, and shorten the time of electric shock [32]. In the same year, it was also shown that intraperitoneal injection of ZS decoction (5.0 g/kg) prolonged the pain threshold in mice [38]. Lee et al. showed that ZS ethanolic extract (100 mg/kg) could significantly ameliorate the scopolamine-induced cognitive impairment in mice [33]. Zhang et al. reported that the water extract of ZS played a necessary role in ameliorating memory retrieval disorders of mice induced by alcohol and improving the learning capacity to some extent [34]. Hou et al. and Wu et al. also got the above results, respectively [35, 36]. Zhu et al. considered ZS jujubosides improved the cognition and memory of Alzheimer's disease rats. The mechanism may be through increase concentration of 5-serotonin content (5-HT), melatonin (MT), melatonin receptor (MTR), and delayed Tau protein hyperphosphorylation [37].

When You et al. compared the sedative and hypnotic effects of *Os Draconis* and ZS decoction (6.25 g/kg), they concluded that ZS could inhibit the spontaneous activity, enhance the rate of falling asleep induced by sodium pentobarbital (in subthreshold dose), prolong the sleep time induced by sodium pentobarbital (in suprathreshold dose), and resist convulsion of mice [39]. Later, ZS was also shown to have an anticonvulsive effect in strychnine nitrate-treated mice [40]. Zhao et al. found that the total alkaloids (50 mg/kg) and cyclopeptide alkaloid (20 mg/kg) from ZS could markedly extend the latent period and dying time of convulsions episode in convulsive mice caused by strychnine [41]. It was noted that jujuboside A (0.05–0.10 g/L) could inhibit the excitatory discharge effect caused by sodium penicillin (in vitro) [42]. In 2003, Zhang et al. showed that jujuboside A (0.1 g/L) could significantly inhibit the release of glutamate in the hippocampus induced by sodium penicillin sodium, and the jujuboside A (0.05–0.10 g/L) could significantly inhibit the glutamate-induced release of intracellular Ca^2+^. These results show that jujuboside A can inhibit glutamate-mediated signaling pathways in the hippocampus [43].

### 5.2. Effect on the Cardiovascular System

Zhang et al. first treated spontaneously hypertensive rats by gavage with ZS total saponins (5 mg/kg) and showed that they had a clear effect on reducing blood pressure [44]. Following this, Liu et al. conducted a similar experiment and observed a blood pressure lowering effect [45]. In addition, Wu et al. found that after orally treating quails with ZS oil (2.5 mL/kg), the total cholesterol (TC), the low density lipoprotein (LDL), and the triglyceride (TG) levels decreased. On the contrary, the high density lipoprotein/the low density lipoprotein (H/L) increased [47]. Wu and Liu et al. also found a similar result in rats and rabbits, respectively [46, 48]. In 1985, Liu et al. first studied the antimyocardial ischemia effect of ZS ethanol extract by intraperitoneal administration (4 g/kg) and intravenous administration (1.5 g/kg). The data showed that the ethanol extract had a tendency to have an antimyocardial ischemia effect in rats injected with pituitrin [51]. In addition, Zhang et al. and Wu showed that ZS total saponins (0.3 g/kg and 0.12 g/kg) could improve myocardial ischemia [49, 50]. Mechanistically, Huang demonstrated that pretreatment with jujuboside A (20 mg/mL) could upregulate Bcl-2 and downregulate Bax protein expression levels, thereby increasing the Bcl-2/Bax ratio, inhibiting the release of cytochrome C from mitochondria, and decreasing the activity of caspase-3 in myocardial tissue, thus reducing cardiomyocyte apoptosis [52].

Experiments in other studies have also shown that ZS has an antiarrhythmic effect. In one experiment, rats were given a sublingual intravenous injection of an ethanol extract of ZS solution (2 g/kg), which was shown to inhibit the arrhythmia induced by aconitine and barium chloride [51]. Following this, Xu et al. comprehensively studied the preventive and therapeutic effects of a water extract of ZS on arrhythmia induced by aconitine, chloroform, and barium chloride in rats and mice; the results showed that the water extract of ZS could treat arrhythmia induced by aconitine and barium chloride in rats and prevent arrhythmia induced by chloroform anesthesia in mice [53]. Additionally, ZS decoction has been shown to improve tolerance to hypoxia [54]. Wu et al. and Zhang et al. studied the antiplatelet aggregation activity of ZS extract. Wu et al. gavaged Japanese male quails with ZS oil (2.5 mg/kg) and Zhang et al. studied the antiplatelet aggregation effect of total saponins (25–660 mg/L) in rabbits. A significant inhibition of platelet aggregation was observed in both Japanese male quails and rabbits, and this effect was closely related to thromboxane B_2_ (TXB_2_) [55].

### 5.3. Sedative and Hypnotic Effects

The sedative and hypnotic effects are the main pharmacological effects of ZS [62]. Wu et al. administered total saponins from ZS at 240 mg/kg to mice, which significantly reduced the spontaneous activity of the mice. At the 480 mg/kg dose, there was a significant increase in the number of mice who lost the reflex induced by a subthreshold dosage of sodium pentobarbital. The same result was obtained when total flavonoids were administered at a dose of 300 mg/kg [57]. You et al. concluded that ZS decoction (6.25 g/kg) could significantly reduce spontaneous activity, prolong the sleep time disrupted by a suprathreshold dose of sodium pentobarbital, and increase the sleep rate in mice disrupted by a subthreshold dose of sodium pentobarbital [39]. Jiang et al. also showed that both ZS flavonoids and saponins (40 mg/kg) could significantly reduce the spontaneous activity of mice and increase the sleeping time of mice. However, ZS polysaccharides did not have the sedative and hypnotic effect [59]. In addition, it was reported that ZS alkaloids could prolong the sleeping time of mice induced by sodium pentobarbital [60]. Jia et al. reported that ZS oil had the same effect; Sun et al. and Li also reported that ZS water extract had the same effect [40, 61, 63].

In 2008, Li et al. studies the sedative and hypnotic effects of ZS oil (1.8 g/kg), obtained by a pressing method and supercritical CO_2_ extraction, in mice. The results showed that both methods could reduce the spontaneous activity of mice, shorten the sleep latency of mice induced by a suprathreshold dose of sodium pentobarbital, and increase the sleep rate in mice induced with a subthreshold dose of sodium pentobarbital [58]. It has also been found that jujuboside A (0.25 mg/mL) could significantly prolong the average sleeping time of *Drosophila* during both the day and night. The dosage of 1 mg/mL was the best concentration and 3 days was the best time for administration [64]. In addition, a mechanistic study by Hu et al. on daytime rapid eye movement *in vitro* and *in vivo* with a methanol extract of ZS showed that the inhibition of neuronal excitation was related to the activation of the *γ*-aminobutyric acid (GABA) system [56]. It was also found that ZS water decoction had sedative and analgesic effects [38].

### 5.4. Antianxiety and Antidepressant Effects

In 2018, Hua gavaged ZS decoction (17.5 g/kg) to rats with anxiety and found that the expression of the c-Fos protein in the basolateral amygdale (BLA) neurons and the discharge frequency of the action potential in neurons were decreased [65]. In 2014, Guo found that the intragastric administration of ZS decoction (10 g/kg) could reduce the abnormal increase in food intake and water intake within 24 hours in rats with Yin deficiency heat syndrome; increase body weight, reduce the kidney and the adrenal function, significantly reducing triiodothyronine (T3) and thyroxine (T4) levels, increasing thyroid-stimulating hormone (TSH) levels; and increase the percentage of open-arm entry times, open-arm retention times, and the number passages through open and dark boxes during combined elevated cross maze and light box tests. From the above findings, it is clear that that ZS has an obvious antianxiety effect in rats with Yin deficiency heat syndrome [66]. Rong et al. have also shown that the antianxiety mechanism of ZS alcohol extract (0.055 g/kg) may be related to an increase in GABA levels in the central nervous system, an increase in GABAAR_1_ expression levels, a decrease in Glu content, and a decrease in NMDAR_1_ expression levels [67]. In addition, another study suggested that the ethanolic extract (0.5 g/kg) possessed anxiolytic effect, and it also possessed sedative effect at higher dose (2.0 g/kg) [68].

The antidepressant effects of ZS have also been studied. Zhang et al. compared the effects of a water decoction and ZS polysaccharides and alkaloids (2.0 g/kg) on depression in mice. The data showed that the water decoction had the best effect followed by that of the alkaloids and polysaccharides [69]. In 2011, Zhao et al. studied the effects of total flavonoids (50, 100, and 200 mg/kg) in a behavioral despair depression model in mice. In all three treatment groups, there was a reduction in forced swimming and tail suspension immobility times [70]. Zhao et al. performed a similar experiment in the same year [71]. Zhu et al. investigated the antidepressant activity of ZS total alkaloids (5, 10, and 20 mg/kg) using depression models such as the reserpine-induced hypothermia test, tail suspension stress test, and open field test. The results showed that alkaloids could shorten the immobility time of tail suspension in mice, and there was a dose-dependent relationship among the three dosage groups. There was an effective antagonism of hypothermia in the mice. The open field experiment showed that there was no significant difference between the three groups in autonomous activity; thus, it was concluded that the antidepressant effect was not caused by an enhancement of autonomous activity [72].

### 5.5. Anti-Inflammatory and Antioxidative Effects

Bao et al. studied the effects of ZS water extract (1 g/100 g) on capillary permeability (abdominal cavity, back, and auricle) by intragastric administration in mice and on egg-white-induced foot swelling and granulation tissue proliferation in rats. The results showed that the ZS water extract could inhibit capillary permeability in the abdominal cavity, the back skin, and the auricle of mice. The anti-inflammatory effects of the extract on egg-white albumin-induced swelling in the hind feet and granuloma induction caused by implantation of paper under the axilla of rats were similar to those of prednisone [73].

Zhang et al. assessed the *in vitro* total antioxidant (TA) capacity, superoxide radical scavenging capacity (O_2_ • ^−^), hydroxyl radical scavenging capacity (• OH), DDPH radical scavenging capacity, and antilipid peroxidation of ZS oil. The results showed that the scavenging ability of ZS oil toward hydroxyl radicals was lower than that of vitamin E (VE), whereas the total antioxidant capacity was equal to that of VE. In contrast, the DPPH radical scavenging ability, superoxide anion radical scavenging ability, and the antilipid peroxidation (ALP) effect were all better than those of VE. Experiments *in vivo* also showed that ZS oil could significantly reduce the malondialdehyde (MDA) content in the blood and liver of a D-galactose oxidation model in mice and significantly increase glutathione peroxidase (GSH-Px) activity [83]. Zhao et al. also found that total flavonoids (0.05 mg/mL) had an obvious scavenging ability for DPPH and ABTS + radicals (IC50 values of 0.70 mg/mL and 0.15 mg/mL, respectively), as well as an ability to reduce potassium ferricyanide [74]. However, these chemical antioxidant assays like the DPPH assay are only simple and useful chemical assays; the therapeutic benefits of ZS were worth further studying. In 2018, Lin et al. reported ZS polysaccharides (5 *μ*g/mL) could significantly promote the proliferation of RAW264.7 cells. Western blot test showed that ZS polysaccharides could significantly promote the release of NO and the expression of stress response protein (COX-2 and iNOS) in RAW264.7 cells. In addition, ZS polysaccharides could significantly promote the phosphorylation of I*κ*B-*α* and ERK protein, which also indicated that the polysaccharide has a certain immunomodulatory effect [77].

### 5.6. Other Pharmacological Effects

In addition to the pharmacological effects described above, ZS has other pharmacological effects. A study showed that oral ZS alcohol extract (5 g/kg) increased the appetite and weight of mice, enhanced the cellular and humoral immune functions (IF), and antagonized the inhibition of delayed hypersensitivity (DH) induced by cyclophosphamide in mice. All these indicate that ZS alcohol extract had an immunopotentiating effect [75]. In 2010, Mishra and Bhatia also indicated that ZS aqueous-ethanolic extract (100–400 mg/kg) had the immunomodulatory potential [76]. Wang et al. examined the effect of ZS oil on body weight and survival time of mice with Ehrlich's ascites carcinoma (*In vivo*). The study proved that ZS oil (0.35 mL/kg) could significantly prolong the survival in mice with cancer and inhibit body weight increases in mice with cancer in later life; it indicated that ZS had obvious antitumor effect on Ehrlich's ascites carcinoma [78]. A separate study showed that ZS water decoction (20 ml/kg) had a protective effect on the decrease in superoxide dismutase (SOD) in febrile mice induced by endotoxin [79]. In addition, Huang's team found that extracts (100 mug/mL) inhibited the growth of human hepatocellular carcinoma cells (HepG2) [80, 81]. In 2020, Yang et al. indicated that the 6‴-feruloylspinosin of ZS (50 *μ*g/mL) has neuroprotective effects. It could reduce beta-amyloid-induced cytotoxicity, lead to increasing a lifespan, and alleviate the oxidative stress. Furthermore, the functions are closely related to the promotion of autophagic activity [82].

In summary, ZS has a wide range of pharmacological activities, which include effects on the central nervous and cardiovascular systems. The main pharmacological effects of ZS are sedation and hypnosis, anti-inflammation, antioxidation, anticonvulsion, blood pressure and lipid lowering, antiplatelet aggregation, enhancing immune function, antihypoxia, antimyocardial ischemia, antiarrhythmia, and antiaging. These findings show that this plant can be used to treat many diseases. Although there are many pharmacological studies using ZS extracts and some of the main components of ZS have been identified, many of their mechanisms are unclear. Therefore, the pharmacological mechanism of action should be further studied.

## 6. Pharmacokinetics

Currently, little research has been performed to assess the pharmacokinetics of extracts and compounds derived from ZS. Liu et al. used LC–MS/MS method to study the pharmacokinetics of jujuboside A (oral administration, 30 mg/kg) in rats and successfully obtained the main pharmacokinetics: Kel = 0.1022 ± 0.023 h^−1^, *t*_(1/2*β*)_ = 6.7 ± 0.9 h, AUC_0⟶36_ = 1989.6 ± 421.7 h·ng^−1^·mL^−1^, AUC_0⟶∞_ = 2159.1 ± 401.4 h·ng^−1^·mL^−1^, Vd = 131.3 ± 38.6 L, Cl_tot_ = 13.95 ± 2.47 h·ng^−1^·mL^−1^, *T*_max_ = 2.0 ± 0.0 h, *C*_max_ = 252.4 ± 39.7 *μ*g·L^−1^ [84]. Zhang et al. used HPLC-MS/MS method to detect the concentration of jujuboside A in rat plasma and study the pharmacokinetics. The pharmacokinetic data was as follows: Ke = 0.28 h, *t*_1/2_ = 2.55 h, AUC_0⟶*t*_ = h·ng^−1^·mL^−1^, AUC_0⟶∞_ = 3201.51 h·ng^−1^·mL^−1^ in rat with intravenous administration and Ke = 0.51 h, *t*_1/2_ = 1.35 h, AUC_0⟶*t*_ = 206.02 h·ng^−1^·mL^−1^, AUC_0⟶∞_ = 211.13 h·ng^−1^·mL^−1^ in rat with oral administration [85]. Wang and Zheng et al. carried out two similar experiments and obtained the same results, respectively [86, 87]. Guo studied the distribution of spinosin, jujuboside A, and jujuboside B in rats after the intragastric administration of a water decoction. These components could be detected in rats within 10 min and were detectable for up to 240 min. The average distribution concentration and the AUC_0-*t*_ of spinosin were the largest in the small intestine, those of jujuboside A were the largest in the lung, and those of jujuboside B were the largest in the large intestine [66]. In 2015, Gao et al. studied the pharmacokinetic effect of different combined administration with ZS and its main components in rats. The results showed that the maximum plasma concentration (*C*_max_) and area under curve (AUC_0-*t*_) of spinosin and ferulic acid in ZS-*Fructus Schisandrae Chinensis* and ZS-*Salviae miltiorrhizae Radix* groups were decreased, and the clearance rate (CL/F) was increased compared with the ZS group. However, the *Zaoren Anshen prescription* group showed higher *C*_max_ and AUC_0-*t*_ for spinosin and ferulic acid, but lower CL/F. Therefore, compared with ZS group, the prescription groups showed slower metabolism of spinosin and ferulic acid and higher bioavailability [88]. Qiao studied the pharmacokinetics of flavonoids from ZS in rats. The results showed that after oral administration, the blood drug concentration of 6‴-feruloylspinosin and 6‴-p-coumaroylspinosin reached a maximum after approximately 45 min; however, the AUC, the clearance rate (Ke), and the half-life (*T*_1/2_), among other parameters, were different. The two compounds followed a first-order kinetics elimination process *in vivo* [89].

In summary, the bioavailability of jujuboside A after intragastric administration was very low compared with intravenous administration; preliminary speculation may be that the numerous drugs are in the form of prototype or present in the gastrointestinal tract after some transformation. In addition, different combinations of drugs have different effects on the pharmacokinetics of ZS and its main components in rats; this may be due to the fact that certain components of different drugs affect the rate of absorption and elimination in the process of drug combination. These speculations require further research to support.

## 7. Toxicology

To date, ZS has been regarded as a traditional Chinese medicine with a high level of safety, and very few toxic reactions have been found in either dietary or clinical applications. According to the *Chinese Materia Medica*, ZS is toxic only at high doses. The dose of 150 g/kg was given to mice by intragastric administration without toxic symptoms. Chronic toxicity test in rats showed that its toxicity was extremely low too. The lethal dose of intraperitoneal injection in mice was 14.33 ± 2 g/kg [8]. Sameena Alam et al. carried out an oral toxicity test on ZS root extract and showed that the extract (2500 mg/kg) had no toxicity or lethal effects in mice [90].

A study exploring the toxicological characteristics of a compound capsule of *Gastrodiae Rhizoma* and ZS found no toxic effects or animal deaths at the maximum dosage (30000 mg) of the sample after gavage. Genetic toxicity tests including the Ames test, the mouse bone marrow cell micronucleus test, and the mouse sperm malformation test all showed negative results. Therefore, the capsule has no genetic toxicity as per the scope of the above tests and thus should be further researched and developed [91]. Acute and subchronic toxicity tests of a ZS oral liquid (500 mg/mL) have also been carried out in chickens. No toxic effects were observed with either dosing regimen. The effect of the oral liquid on blood biochemical indexes was reversible in the subchronic toxicity test; thus, continuous oral administration was found to be safe in chickens [92].

Wang et al. injected different doses of an alcohol extract of ZS into the caudal vein of mice and found that some mice had a toxic reaction and died. The LD_50_ was 27.5 g/kg with 95% confidence limits of 25.1–30.1 g/kg. An autopsy of the d animals showed no pathological changes in their main organs. However, no mice died in 14 consecutive days of observation after gavage administration of 340 g/kg of the same alcohol extract of ZS [93]. Therefore, ZS is considered a safe traditional Chinese medicine.

## 8. Future Perspectives and Conclusions

In summary, ZS is a traditional Chinese medicine especially used in Asian countries. Presently, numerous chemical constituents have been isolated and identified from this plant. Several experts have studied the plant and made significant contributions in several areas. However, new problems and challenges remain in the research of it. In particular, we need further research and exploration to meet clinical requirements.

First, as a traditional Chinese medicine, although ZS has been studied extensively in recent years, the research has mainly concentrated on saponins, flavonoids, alkaloids, and so on; the actions of fatty oils and other chemical components are rarely reported. However, some fatty oils also have good pharmacological activity. For example, ZS oil has good antioxidant properties, and it is one of the important material bases to reflect the efficacy of ZS [17]. Therefore, the development of ZS oil products has broad application prospects. Second, few studies have been performed to assess pharmacokinetics and toxicity. Researchers should pay more attention to this aspect. Researchers should also assess toxicity *in vivo* to improve drug safety, to allow for further drug development that should lay the foundation for future clinical drug use. In China, there is a shortage of supply in the market as the wild ZS sources are decreasing each day, and thus, some fake plants, such as *Lizaoren, ZhiqiZi, Dianzaoren,* and *Bingdou,* have appeared in the pharmaceutical market [94]. In addition, the shapes of some fruits are very similar to that of ZS, and thus, it is difficult to distinguish them by traditional identification methods. Recently, a PCR method based on the Internal Transcribed Spacer (ITS) sequence site has been developed that can differentiate ZS from its fake counterparts. This method uses primer ZmITS3 to amplify a 66 bp fragment from ZS, but no such band can be amplified from the fake plants. Thus, ZmITS3 can be used as a specific primer to identify genuine ZS and we need further research in this aspect to improve the quality of medicinal materials [95]. Fourth, a growing number of people are suffering from insomnia and anxiety with the increasing pressure on people's life and work. However, the long-term use of sleeping pills not only impairs memory function and induces Alzheimer's disease but also leads to addiction and drug dependence. Patients who understand the concept of preventive medicine may be willing to move from drug treatment toward the use of functional foods derived from natural plant resources. ZS, as an important medicine for nourishing and calming the mind, could therefore be used as an alternative to prevent and treat insomnia.

This paper has systematically and comprehensively introduced the research status of ZS in recent years, including its botanical, and its traditional uses, phytochemical, pharmacological, pharmacokinetic, and toxicological characteristics. Although great progress has been made, several aspects are yet to be known.

We hope that this paper can provide some suggestions for future research directions of traditional Chinese medicine.

## Figures and Tables

**Table 1:** Pharmacological effects of ZS.

Pharmacological effects	Detail	Extracts/compounds	Results	In vitro/in vivo	Ref.
Effect on the central nervous system	Improving dysmnesia	Flavonoids	Latent period↑, erroneous times↓, time of electric shock↓	In vivo	[32]
		Ethanolic extract	Passive avoidance task↑, Y-maze task↑, Morris water maze task↑, ERK-CREB-BDNF↑, NMDA↑	In vivo	[33]
		Water extract	Passive avoidance task↑, Y-maze task↑	In vivo	[34]
		Decoction	Step down test↑, Morris water maze task↑	In vivo	[35]
		ZS oil	Passive avoidance task↑, Morris water maze task↑	In vivo	[36]
		Jujubosides	5-HT↑, MT↑, MTR↑, Tau protein↓	In vivo	[37]
	Analgesic effects	Water extract	Pain threshold↑	In vivo	[38]
	Analgesic effects	Decoction	Spontaneous activity↓; sleep time↑; convulsion; ↓	In vivo	[39, 40]
		Total alkaloids, cyclopeptide and alkaloid	Latent period of convulsion↑; dying time↑	In vivo	[41]
		Jujuboside A	Hyperactivity of hippocampal CA1 area↓	In vitro	[42]
		Jujuboside A	Release of glutamate and intracellular Ca^2+^↓	In vivo	[43]
Effect on the cardiovascular system	Antihypertensive effects	Total saponins	Tail artery blood pressure↓	In vivo	[44, 45]
	Antihyperlipoidemia effects	Total saponins	TC, LDL, TG ↓; H/L↑	In vivo	[46]
		ZS oil	TC, LDL, TG ↓; H/L↑	In vivo	[47, 48]
	Improving myocardial ischemia	Total saponins	Infarcted area↓, S-T piece and T wave value↓	In vivo	[49]
		Total saponins	T wave value↓	In vivo	[50]
		Ethanol extract	Myocardial ischemic rats↓	In vivo	[51]
	Alleviating myocardial reperfusion injure	Jujuboside A	Bcl-2/Bax↑, cytochrome C↓, caspase-3↓	In vitro	[52]
	Antiarrhythmic effects	Water extract	Occurrence of Arrhythmic↑, mouse survival time↑, ventricular fibrillation↓	In vivo	[53]
		Ethanol extract	Recovered sinus rhythm time↓	In vivo	[51]
	Improving hypoxia tolerance	Decoction	Oxygen consumption of brain tissue↓	In vivo	[54]
	Antiplatelet aggregation	Total saponins	Platelet aggregation↓, TXB2↓	In vitro	[55]
		Methanol extract	GABAA receptor subunits and glutamic acid decarboxylase↑	In vivo	[56]
Sedative and hypnotic effects	Prolonging the sleep time	Total saponins, total flavonoids	Spontaneous activity↓, sleeping rate↑	In vivo	[57]
		ZS oil	Spontaneous activity↓, sleeping time↑, sleeping number↑, sleeping latency↓	In vivo	[58]
		Flavonoids and saponins	Spontaneous activity↓, sleeping time↑	In vivo	[59]
		Alkaloids	Sleeping time↑	In vivo	[60]
		Decoction	Spontaneous activity↓, sleep time↑, convulsion; ↓	In vivo	[39]
		ZS oil	Sleeping rate↑, sleeping time↑	In vivo	[61]
		Water extract	Sleeping time↑	In vivo	[62]
		Total extract	Spontaneous activity↓, sleeping time↑	In vivo	[63]
		Jujuboside A	Total time of sleep during the day and night↑	In vitro	[64]
		Water extract	Activity times↓, stand-up times↓	In vivo	[38]
Antianxiety and antidepressant effects	Antianxiety effects	Decoction	c-Fos protein↓	In vivo	[65]
		Decoction	Food and water intake↓, weight↑, T3↓, T4↓, TSH↑	In vivo	[66]
		Alcohol extract	GABA↑, GABAAR1↑, Glu↓, NMDAR1↓	In vivo	[67]
		Alcohol extract	Sleeping time↑, locomotor activity↓	In vivo	[68]
	Antidepressant effects	Decoction, polysaccharide, alkaloid	Immobility time↓, SOD↑, MDA↓	In vivo	[69]
		Total flavonoids	Immobility time↓	In vivo	[70]
		Total saponins	Immobility time↓	In vivo	[71]
		Total alkaloids	Immobility time↓	In vivo	[72]
Anti-inflammatory and antioxidant effects	Anti-inflammatory effects	Water extract	Capillary permeability↓, foot swelling↓, granulation tissue proliferation↓	In vivo	[73]
	Antioxidant effects	ZS oil	TAC↑, O_2_•^−^↑, •OH↑, DDPH↑, ALP↑, MDA↓, GSH-Px↑	In vitro	[64]
		Total flavonoids	Scavenging ability for DPPH and ABTS + radicals↑, reduce potassium ferricyanide↑	In vitro	[74]
Other pharmacological effects	Immunomodulatory activity	Ethanol extract	Appetite↑, body weight↑, IF↑, DH↓	In vivo	[75]
		Ethanol extract	Interferon-gamma↑, interleukin-4↑	In vivo	[76]
		Polysaccharide	NO↑, COX-2↑, iNOS↑, p-I*κ*B-*α*↑, p-ERK↑	In vitro	[77]
	Preventing and treating of cancer	ZS oil	Body weight↓, survival time↑	In vivo	[78]
	Antiaging effects	Decoction	SOD↑	In vivo	[79]
	Protecting kidney	Flavonoid suspension	HepG2↓	In vitro	[80, 81]
	Neuroprotective effects	6‴-Feruloylspinosin	ROS↓, p62↓, pink1/parkin ↑	In vitro	[82]
